# Identification of Essential Oils Including Garlic Oil and Black Pepper Oil with High Activity against *Babesia duncani*

**DOI:** 10.3390/pathogens9060466

**Published:** 2020-06-12

**Authors:** Yumin Zhang, Chunxiang Bai, Wanliang Shi, Hector Alvarez-Manzo, Ying Zhang

**Affiliations:** Department of Molecular Microbiology and Immunology, Johns Hopkins Bloomberg School of Public Health, Johns Hopkins University, Baltimore, MD 21205, USA; yzhan424@jhu.edu (Y.Z.); cbai4@jhmi.edu (C.B.); wshi3@jhu.edu (W.S.); halvare3@jhu.edu (H.A.-M.)

**Keywords:** *Babesia duncani*, essential oil screen, garlic oil, black pepper oil, diallyl disulfide (DADS), β-caryophyllene (BCP)

## Abstract

Some evidence indicated that human babesiosis caused by *Babesia duncani* has spread widely in North America. However, current therapeutic regimens (atovaquone + azithromycin) for human babesiosis are suboptimal with frequent recrudescence and side effects, and furthermore, there is no specific treatment for human babesiosis caused by *B. duncani*. Here, we screened 97 essential oils and identified 10 essential oils (garlic, black pepper, tarragon, palo santo, coconut, pine, meditation, cajeput, moringa, and stress relief) at a low concentration (0.001%; *v*/*v*) that showed good inhibitory activity against *B. duncani* in the hamster red blood cell culture model. Among them, garlic oil and black pepper oil performed best, as well as their potential active ingredients diallyl disulfide (DADS) and β-caryophyllene (BCP), respectively. Interestingly, further subculture study indicated that *B. duncani* could relapse after treatment with current therapeutic drugs atovaquone or azithromycin even at high concentrations. In contrast, the combination of garlic oil or DADS and azithromycin showed eradication of *B. duncani* at low concentrations without regrowth. These results are encouraging and suggest that the garlic-derived sulfur compound DADS and β-caryophyllene (BCP) may be promising drug candidates for evaluation of their ability to cure persistent *B. duncani* infections in the future.

## 1. Introduction

Human babesiosis is an emerging tick-born disease caused by members of the genus *Babesia*, which are similar to *Plasmodium* parasites that cause malaria as both of them are intraerythrocytic protozoan parasites. *Babesia divergens*, *Babesia microti,* and *Babesia duncani* cause the most human babesiosis cases worldwide [1]. Babesiosis is mostly transmitted by tick bite and also by blood transfusion and organ transplantation [2,3]. The clinical symptoms of babesiosis patients can range from flu-like mild to malaria-like severe symptoms, and patients who undergo immune disease (e.g., acquired immune deficiency syndrome (AIDS)), immunosuppressive therapies, and splenectomy can suffer more severe manifestations, and even death [1]. To date, cases of human babesiosis in the USA have been increasing in the past two decades, and babesiosis was added to the list of nationally notifiable diseases by the Centers for Disease Control and Prevention (CDC) in 2011 [4].

In the United States, *B. microti* and *B. duncani* are commonly recognized and identified in humans. Even though *B. duncani* was firstly described in Washington State as strain WA1 in 1991 [5], a total of 1119 cases infected by *B. duncani* were identified in the U.S. and Canada during 2011–2017 [6], indicating that *B. duncani* had been detected and diagnosed widely in North America. Although intraerythrocytic stages of *B. duncani* are morphologically indistinguishable from *B. microti*, based on the phylogenetic analysis (*18S RNA* gene, *ITS2* gene, *Cyt b* gene, and *Cox I* gene) *B. duncani* n. sp. are in a distinct clade separated from *B. microti* [7,8]. According to the treatment experience of physicians, *B. duncani* was harder to treat than *B. microti*, and patients typically required longer anti-babesiosis treatment [6]. Moreover, in animal models, *B. duncani* can give rise to more severe clinical and hematological presentation and then eventually death in hamsters and mice [9].

To date, drugs and drug combinations are still the predominant weapons to treat human babesiosis due to the absence of vaccines. The current treatments for babesiosis are drug combinations of atovaquone plus azithromycin or clindamycin plus quinine [10]. In some cases, babesia infections can be refractory, and recrudescence of infection may occur after therapy, even in persistence for more than two years [11,12]. Furthermore, clinical studies revealed that both treatment regimens are associated with significant adverse effects, and in the group treated with clindamycin + quinine, a much higher percentage suffered adverse effects than those who were treated with atovaquone + azithromycin (72% vs. 15%) [9]. These deficiencies emphasize the need for more effective therapies that target persistence while having fewer side effects.

*B. duncani* culture can be established continuously and long-term ex vivo in hamster or human erythrocytes [1,13,14], whereas only short-term culture can be achieved with *B. microti*. Hence, *B. duncani* is an appropriate species of choice for convenient drug screens for developing more effective human babesiosis treatment. However, there is no literature that reported results of drug screens on *B. duncani* in vitro or in vivo so far. Furthermore, Abraham et al. (2018) revealed that, in human erythrocytes, *B. duncani* showed high tolerance to routine therapies [14]. Thus, it is necessary to develop new therapeutic options for human babesiosis.

Essential oils containing volatile chemical compounds extracted from plants are widely used in aromatherapy, food reservation, and also potentially in medical therapy, especially with recent concerns about antibiotic resistance [1,15,16]. Certain essential oils have been discovered that have antibacterial activity against multidrug-resistant Gram-negative clinical isolates [17]. In our previous studies, we have identified many essential oils with good activity against *Borrelia burgdorferi* and *Bartonella henselae*, which usually can cause co-infections with *Babesia* sp. through tick transmission [18,19]. In this study, we screened a panel of 97 essential oils for activity against *B. duncani* and identified some promising active hits. We showed that some active hits in combination with other drugs to be more potent than the current treatment for human babesiosis.

## 2. Results

### 2.1. In Vitro Screening of Essential Oils for Inhibitory Activity against B. duncani

In our preliminary experiment, we determined that essential oils at a concentration higher than 0.001% (*v*/*v*) did not cause significant lysis of the hamster erythrocytes within three days. However, most essential oils at 0.010% or higher could cause lysis of erythrocytes in 6 h. Therefore, we evaluated a panel of 97 essential oils at this concentration of 0.001% (*v*/*v*) for inhibitory activity against *B. duncani* at an initial parasitemia of 2% in the 96-well plates which were incubated for 72 h. We found 10 essential oils including garlic, black pepper, tarragon, palo santo, coconut, pine, meditation, cajeput, moringa, and stress relief that showed more than 50% inhibitory effects at 0.001% against *B. duncani* (Table 1).

**Figure 1:**
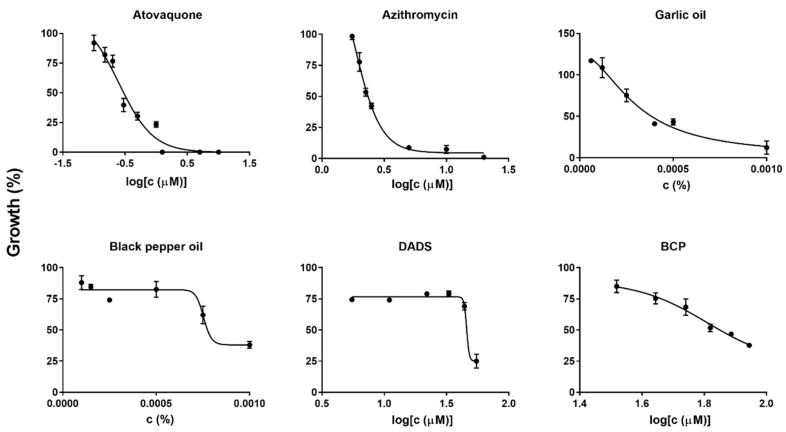
Evaluation of in vitro drug and essential oil susceptibility of *Babesia duncani* to atovaquone, azithromycin, garlic oil, black pepper oil, diallyl disulfide (DADS), and β-caryophyllene (BCP) at different concentrations: atovaquone (μM): 0.10, 0.15, 0.20, 0.30, 0.50, 1.0, 1.2, 5.0, 10; azithromycin (μM): 1.7, 2.0, 2.2, 2.5, 5.0, 10, 20; garlic oil (%): 0.000060, 0.00012, 0.00025, 0.00040, 0.00050, 0.0010; black pepper (%): 0.00010, 0.00015, 0.00025, 0.00050, 0.00075, 0.0010; DADS (μM): 5.5, 11, 22, 33, 44, 55; BCP (μM): 33, 44, 55, 66, 77, 88. SYBR Green I assay was performed at 72 h after drug or essential oil exposure. Each drug or essential oil concentration was made in triplicate. The infected red blood cells (RBCs) treated with 100 μM atovaquone were set as 0% growth, and wells with infected erythrocytes in drug-free medium and 1% DMSO vehicle were set as 100% growth. GraphPad Prism (version 7.0) (GraphPad Software, San Diego, CA, USA) was used to generate dose–response curves by fitting a nonlinear regression curve to the data. Abbreviation: c, concentration.

Among them, garlic oil and black pepper oil showed the highest activity against *B. duncani* growth. Dose–response assays identified the half maximal inhibitory concentration (IC_50_) values of garlic oil and black pepper oil to be 0.00030% and 0.00075%, respectively (Figure 1). In our previous study, we found some essential oils may have autofluorescence and would neither dissolve in DMSO nor culture medium [18], but these problems were no longer present when the 100–1000-fold lower concentrations that were used in this study. The results indicated that some essential oils indeed have anti-*Babesia* activity ex vivo, and garlic and black pepper essential oils had the most potent activity against *B. duncani* in this study.

### 2.2. Diallyl Disulfide (DADS) and Β-Caryophyllene (BCP) as Highly Potent Active Ingredients of Garlic Oil and Black Pepper Oil against B. duncani Growth In Vitro

Given garlic oil and black pepper oil were the best anti-*Babesia* essential oils in our primary screening, we also tested their potential active ingredients, diallyl disulfide (DADS) and β-caryophyllene (BCP). DADS is the major constituent of garlic oil. BCP exists in many essential oils, such as black pepper, cloves, oregano, rosemary, and ylang-ylang, and these essential oils indeed showed inhibitory efficacy in this study (Table S1). The approximate quantity range of BCP in different black pepper cultivars is 7–38% [20]. Interestingly, DADS and BCP showed similar high activity against *B. duncani*, and the IC_50_ values were 0.00084% (6.7 μg/mL; 46 µM) and 0.0014% (13 μg/mL; 64 μM), respectively. The values were a little bit higher than those of their corresponding essential oils. For comparison, we additionally tested the front-line clinical drugs atovaquone and azithromycin in our drug testing system. The IC_50_ values of atovaquone and azithromycin were 0.25 μM (0.092 μg/mL) and 1.8 μM (1.3 μg/mL), respectively (Figure 1), which is comparable to their results when tested in vitro in human erythrocytes [14].

### 2.3. Morphological Changes of Treated B. duncani

**Figure 2:**
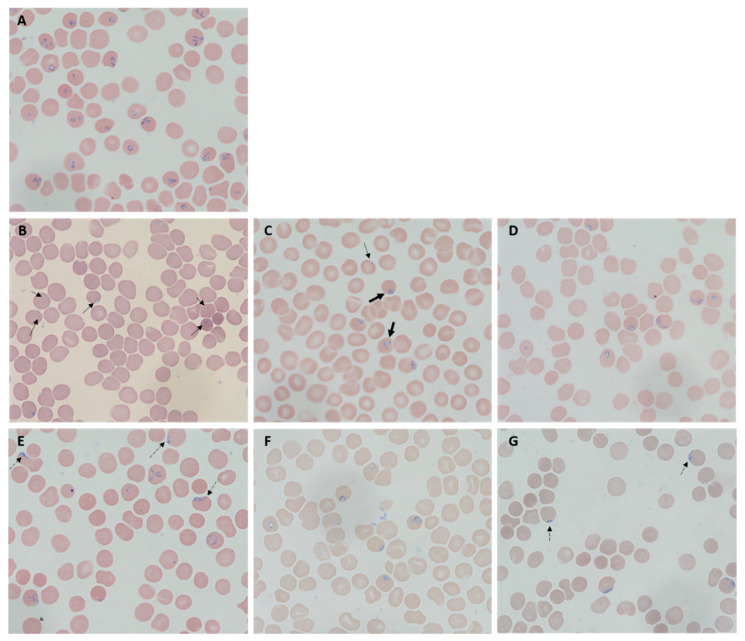
Morphology of *Babesia duncani* observed after treatment of atovaquone (**B**), azithromycin (**C**), garlic oil (**D**), black oil (**E**), diallyl disulfide (**F**), and β-caryophyllene (**G**) at their respective concentrations of 2× IC_50_ value compared to untreated control (**A**) after 72 h exposure. The initial parasitemia of 2% was used for treatments. Thin arrows indicate dot or granule parasites; bold arrows indicate teratogenic parasites; dash arrows indicate elongated strand parasites located near the erythrocytic rim.

Morphology of *B. duncani* observed after treatment of atovaquone, azithromycin, garlic oil, black pepper oil, DADS, and BCP at their respective concentrations of 2× IC_50_ value was compared with untreated control after 72 h exposure (Figure 2). *B. duncani* treated by atovaquone appeared to have plenty of dot or granule forms with tiny cytoplasm and condensed chromatin (Figure 2B), and that treated by azithromycin showed ugly, disintegrated, and teratogenic parasites (Figure 2C). Both black pepper oil- and BCP-treated *B. duncani* changed to elongated strand form located near the rim of erythrocytes (Figure 2E,G), while the morphology of *B. duncani* treated by garlic oil and DADS (Figure 2D,F) did not show significant difference from that of the untreated one (Figure 2A).

### 2.4. Subculture Studies to Evaluate the Viability of B. duncani Treated with Atovaquone, Azithromycin, Garlic Oil, DADS, and Their Combinations 

**Figure 3:**
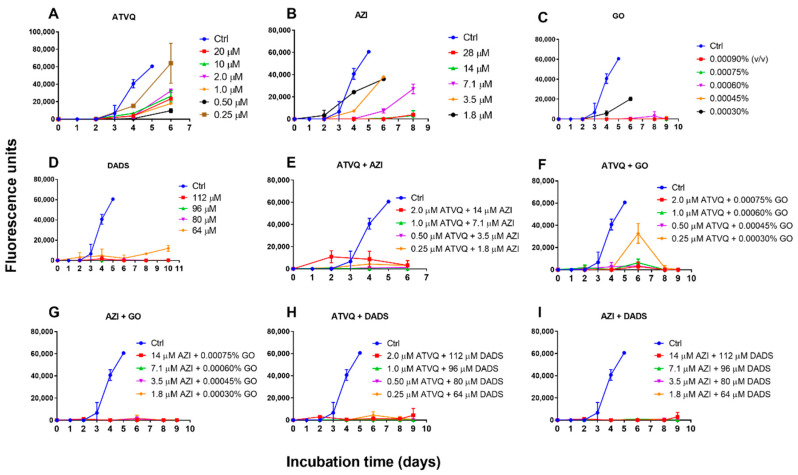
Subculture of *B. duncani* in hamster erythrocytes after 72 h treatment with atovaquone (**A**), azithromycin (**B**), garlic oil (**C**), diallyl disulfide (**D**), atovaquone plus azithromycin (**E**), atovaquone plus garlic oil (**F**), azithromycin plus garlic oil (**G**), atovaquone plus diallyl disulfide (**H**), and azithromycin plus diallyl disulfide (**I**) at different concentrations in 96-well plates. The growth of the subculture was examined by SYBR Green I stain, and the fluorescence units of day 0 were normalized as 0. Abbreviation: ATVQ, atovaquone; AZI, azithromycin; GO, garlic oil; DADS, diallyl disulfide.

To further confirm the activity of garlic oil and DADS in killing *B. duncani*, we performed subculture studies by re-inoculating treated infected erythrocytes into fresh uninfected erythrocytes to monitor regrowth. For comparison, we also validated the activity of atovaquone and azithromycin, and their combination with garlic oil or DADS. We found that garlic oil- and DADS-treated *B. duncani* can regrow at 1× IC_50_ value, but no growth occurred after treatment at 1.25×, 1.50×, and 1.75× IC_50_ value (Figure 3C,D). In contrast, *B. duncani* could regrow after atovaquone treatment of 1×, 2×, 4×, 8×, 40×, and 80× the IC_50_ value, and after azithromycin treatment of 1×, 2×, 4× the IC_50_ value (Figure 3A,B). Obvious regrowth did not occur after azithromycin treatment of 8× and 16× the IC_50_ value at day 8 when erythrocytes began to lyse by the end of its life span (Figure 3B). Interestingly, the combination of atovaquone at 8× IC_50_ value and azithromycin at 8× IC_50_ value-treated *B. duncani* regrew at the beginning, reached peak at day 2, and then declined (Figure 3E). Treatment by a lower concentration of the combination of atovaquone (1×, 2×, 4× the IC_50_ value) and azithromycin (1×, 2×, 4× the IC_50_ value) did not give rise to obvious regrowth (Figure 3E). Atovaquone + garlic oil or DADS combination and azithromycin + garlic oil or DADS combination showed good activity to block *B. duncani* regrowth after respective treatment, except for the combination of 1× IC_50_ value atovaquone plus 1× IC_50_ value garlic oil, which led to regrowth at day 4 and reached peak at day 6 (Figure 3F–I).

## 3. Discussion

Babesiosis has been classified as a nationally notifiable disease since 2011 and is recognized as an emerging health risk in several parts of the world [21]. The actual number of cases caused by *B. microti* and *B. duncani* are supposed to be much greater because many are undetected and unreported, and some *B. duncani* cases will be in the asymptomatic phase [6,22]. However, the current treatment options using atovaquone, azithromycin, clindamycin, quinine, and their combinations for human babesiosis are suboptimal as they were suggested based on their antimalarial activity, and these regimens are associated with significant side effects and treatment failures. On the other hand, no specific treatment has been proposed for babesiosis caused by *B. duncani*, even though *B. duncani* has been proved to have many unique characteristics in phylogeny, animal model, and clinical manifestations [6,7,9]. Thus, it is necessary to find more effective and specific treatments for *B. duncani*-related babesiosis. Our study demonstrated that from a screening of 97 essential oils, 10 essential oils (garlic, black pepper, tarragon, palo santo, coconut, pine, meditation, cajeput, moringa, and stress relief) had good activity in vitro against *B. duncani* growth in vitro. In particular, garlic oil and black pepper oil, showed remarkably high activity at much lower concentrations than those reported for most essential oils in terms of their antibacterial activity [23,24].

Both garlic oil and black pepper oil are traditional diet food supplements across the world. Garlic has been used in herbal medicine for thousands of years, and fresh garlic has been proved to have wide spectrum of antimicrobial activities against many bacteria, fungi, and viruses [25,26,27,28]. In this study, garlic oil was the top hit among 97 essential oils against the growth of *B. duncani* at a concentration that was extremely lower than that against bacterial growth in previous reports [29,30], but comparable to that of allicin against other parasites, such as *Plasmodium* and *Entamoeba* [31,32]. Salama et al. indicated that allicin had inhibitory effect in vitro on *Babesia bovis*, *Babesia bigemina*, and *Babesia caballi*, but the IC_50_ values of allicin were quite high (470–818 μM) [33]. This indicated that allicin might not be the most active anti-*Babesia* constituent in garlic. Allicin is the compound that contributes to antibacterial activity in crushed garlic clove, a part of unstable allicin yielded by self-condensation of sulfenic acid molecules then transformed into diallyl sulfide (DAS), DADS, and diallyl trisulfide (DATS) [34]. DADS and DATS are the major constituents of garlic oil, and DADS is reported to comprise about 60% of garlic oil [26]. In our study, DADS showed similar considerable inhibitory effect as garlic oil, at a slightly higher IC_50_ value than that of garlic oil (0.00084% vs. 0.00030%) and may attribute to the activity of other sulfides (DAS and DATS) contained in garlic oil as well. Previous studies have reported that black pepper essential oil and its major compounds terpenoids had potential antibacterial activity [35,36]. To our best knowledge, the antiparasite activity of black pepper identified in this study was not previously reported. A study indicated that black pepper extract showed good inhibitory activity against *Blastocystis hominis* in vitro at the concentration of 100 μg/mL [37]. In this study, we found black pepper essential oil had an in vitro IC_50_ value of 0.00075% concentration against *B. duncani*, and the value of its major terpenoid BCP was 0.0014% (13 μg/mL). It demonstrated that BCP as an active continent of black pepper essential oil has incredible inhibitory efficacy against *B. duncani* and may also explain the inhibitory efficacy of other essential oils including cloves, oregano, rosemary, and ylang ylang, which contain BCP, in our screening.

Previous clinical study indicated *Babesia* infections can be refractory to drug treatment, and recrudescence or relapse of infection may occur after current treatment and can persist even for more than two years [11,12]. In an immunodeficient mouse model, Lawres et al. have proved that current treatment with azithromycin up to 50 mg/kg, clindamycin up to 50 mg/kg, or quinine up to 100 mg/kg had no significant effect on *B. microti*, only atovaquone showed potent activity against *B. microti* during treatment period, but recrudescence appeared in a few days after treatment [38]. Atovaquone also showed potent activity against *B. bovis* (18 nM; IC_50_) and *B. divergens* (32 nM; IC_50_) [39]. However, in a human erythrocyte culture system, Abraham et al. have reported that *B. duncani* revealed unusually high tolerance to the current recommended therapies [14]. These results are consistent with our findings with *B. duncani*, which demonstrated that atovaquone had incredible inhibitory effect at relatively low concentration (0.25 μM), but relapse was observed in subculture test after up to 20 μM atovaquone exposure for 72 h. We also confirmed the better killing effect of *B. duncani* by atovaquone plus azithromycin combination than their monotreatment, but relapse could still transiently arise when they were combined in high dose for unclear reasons. In contrast, in this study *B. duncani* did not regrow after garlic oil and DADS treatment at concentrations that did not lyse host cells, except for the concentrations of the 1× IC_50_ values. In the combination tests, the killing activity of atovaquone plus azithromycin can be improved when one of them was replaced with garlic oil or DADS. Interestingly, as a pure compound, DADS showed better eradication effect than garlic oil, whether used singly or combined with atovaquone or with azithromycin. Our results imply that in light of the good anti-*B. duncani* activity of DADS, garlic oil is a promising alternative to treat human babesiosis when used alone or in combination with azithromycin, considering resistance to atovaquone may emerge as it has been emerging rapidly in malaria treatment.

An important finding of this study is that both garlic oil and DADS plus azithromycin at doses as low as 1× their IC_50_ value resulted in total eradication with no relapse, compared with monotreatment of atovaquone or azithromycin and their combination. Treatment failure of antimicrobial drugs is a serious global health threat, and the non-replicating but viable bacterial cells with drug tolerance were firstly termed “persisters” by Joseph Bigger in 1944 [40]. Besides bacteria, eukaryotic cells such as fungi and parasites can also enter into this dormant phase of drug-tolerant persister-like phenotype [41,42]. Malaria may recrudesce years after completion of therapy because of the persister-like cells hypnozoites of *Plasmodium ovale* or *Plasmodium vivax* hiding in the liver, or small non-replicating ring-stage parasites of *Plasmodium falciparum* persisting in erythrocytes induced by artemisinin derivatives during treatment [43,44]. For *Babesia*, no such extraerythrocytic stage has been described, however, we also found many “parasite dots” of *B. duncani* with reduced cytoplasm and condensed nucleus under microscope after atovaquone exposure, and merozoite and tetrad form was hard to find (Figure 2B). These dormant persister-like parasites may explain the recrudescence of *B. duncani* even after high-dose atovaquone treatment. In contrast, after the exposure in garlic oil or DADS, the morphology of *B. duncani* did not show significant difference from that of the control group, as both merozoites and trophozoites were clearly observed (Figure 2D,F). DADS and other garlic-derived sulfur compounds have been demonstrated to have anticancer effects in vitro and in vivo with some potential mechanisms including inducing cell cycle arrest, growth inhibition, differentiation, and apoptosis [45]. Because the *Babesia* parasite is a eukaryotic cell that also has these cellular processes as a cancer cell, they may share equivalent response mechanisms to DADS and other garlic-derived sulfur compounds. Strong evidence is needed in the future to explain why *B. duncani* was extremely susceptible to garlic oil and DADS. However, it is worth noting that both garlic oil and DADS even at the low concentration studied in our current culture system can significantly reduce the lifespan of hamster erythrocytes in vitro, whether the adverse effect occurs in vivo needs to be addressed in future. In addition, longer subculture study is hampered by the limited lifespan of erythrocytes in this culture system if no fresh erythrocytes are added, indicating that a more effective and convenient approach for determining the viability of *Babesia* needs to be developed.

It has been reported that garlic oil presented direct toxic effects in high dose in mouse and rat models [46]. The median lethal dose (LD_50_) of garlic extract is higher than 30 mg/kg in rodents [47]. DADS is toxic at the dose of 400 mg/kg but well tolerated at the dose of 200 mg/kg in rats [48]. In humans, the reported maximum of tolerated dose of garlic extract was 25 mL [49]. In vitro experiment indicated that active oxygen generated by these sulfides derived from garlic and onions could cause oxidative damage to erythrocytes [50]. Thus, it is necessary to determine whether the anti-babesiosis therapeutic effect presents without side effect in vivo. However, Hiroyuki et al. demonstrated that DADS administrated by intraperitoneal injection at the dose of 1 or 2 mg three times a week was effective in inhibiting the growth of breast cancer cells without serious side effects [37]. BCP is generally recognized as safe (GRAS) by the Flavor and Extract Manufacturers Association, and a 90 day oral gavage study at the dose up to 700 mg/kg/day in rats confirmed the safety of BCP used in medical foods [51]. Interestingly, both garlic oil and BCP could form inclusion complexes with β-cyclodextrin to improve their physicochemical stability and bioavailability [52,53]. Future studies are needed to test the efficacy of DADS and BCP in eradicating *B. duncani* infection in animal models.

In summary, we identified 10 essential oils that showed good inhibitory activity against *B. duncani* in vitro at 0.001% (*v*/*v*). Among them, garlic oil and black pepper oil performed best, as well as DADS and BCP, which are their potential active constituents, respectively. We further demonstrated that the combination of garlic oil or DADS and azithromycin could eradicate *B. duncani* at low concentrations in vitro. Future studies are needed to test the activity of DADS and other garlic-derived sulfur compounds alone and in combination with current drugs to eradicate babesiosis in animal models and their activity against related *Babesia* parasites, and potential mechanisms of action of these compounds against *Babesia* parasites.

## 4. Materials and Methods

### 4.1. Hamster Donor Blood

Hamster whole blood was collected from Golden Syrian hamsters (Charles River) by cardiac puncture using phosphate buffered saline (PBS) containing 15 mM EDTA as anticoagulant according to protocols approved by the Johns Hopkins Institutional Animal Care and Use Committee. Blood was washed three times by centrifugation at 500× *g* for 15 min at 4 °C in PBS, 15 mM EDTA solution with careful removal of the supernatant and buffy coat at each wash. After the last wash, red blood cells (RBCs) were resuspended at a concentration of 50% hematocrit in Puck’s saline glucose buffer with extra glucose (20 g glucose/L) and stored at 4 °C less than 2 weeks before use.

### 4.2. Babesia duncani Culture In Vitro

*Babesia duncani* strain WA1 (ATCC^®^ PRA-302™) was cultured in vitro in HaRGM medium as described by Abraham et al. (2018) with slight modifications [14]. Briefly, 500 μL of cryopreserved culture was used to infect hamsters through intraperitoneal injection and monitor blood parasitemia every day. When the parasitemia reached approximately to 10%, blood was collected by cardiac puncture after isoflurane anesthesia. Washed, infected RBCs (10 μL) were mixed with 90 μL of fresh hamster uninfected RBCs and resuspended in 1.2 mL warm HaRGM and then dispensed into a single well of a 24-well plate. Specifically, 1.2 mL of medium had a depth of 0.7 cm in the 24-well plate for generating a microaerophilic condition [54]. The plate was inoculated at 37 °C under an atmosphere of 5% CO_2_ with 95% humidity. The overlying medium was removed and replaced with fresh HaRGM daily. When the RBCs laid on the bottom turned dark and black, 10 μL of infected RBCs of which parasitemia had been confirmed by Giemsa stain was mixed with 90 μL of fresh uninfected RBCs then cultivated into a new well of the 24-well plate.

### 4.3. In Vitro Evaluation of Essential Oils and Other Chemicals on Inhibition of B. duncani

A total of 97 commercially available essential oils were purchased from Plant Therapy (Twin Falls, ID, USA), Natural Acres (St. Louis, MO, USA), or Plant Guru (Plainfield, NJ, USA) and were used as we described previously [18,19]. Essential oils were prepared in 0.1% (*v*/*v*) stock solution in DMSO then added to 96-well plates containing 200 μL of infected hamster RBCs at 2% parasitemia and 2.5% hematocrit each well to obtain final concentrations of 0.001%. Cultures were incubated at 37 °C for 72 h without replacing medium in a chamber with an atmosphere of 5% CO_2_ with 95% humidity. Then 2 μL of RBCs sediments were removed into 100 μL of lysis buffer consisting of 20 mM Tris, pH 7.4, 5 mM EDTA, 0.008% saponin, 0.08% Triton X-100, and 2× SYBR Green I (10,000× stock, Invitrogen) in 96-well plates. The plates were inoculated at 37 °C in the dark for 60 min followed by plate reading at excitation wavelength at 490 nm and a fluorescence intensity at 520 nm in microplate reader (HTS 7000 plus Bio Assay Reader, PerkinElmer Inc., Waltham, MA, USA). We selected those essential oils that showed more than 50% inhibitory effect at 0.001% (*v*/*v*) after 72 h exposure for a further IC_50_ susceptibility assay. For the IC_50_ susceptibility assay, *B. duncani* WA1 cultures were exposed to increasing concentrations of garlic oil, black pepper oil, diallyl disulfide (DADS), β-caryophyllene (BCP), atovaquone, azithromycin, and their combinations. Each IC_50_ was performed in triplicates in 96-well plates with a starting 2% parasitemia and 2.5% hematocrit in 200 μL of culture medium followed by SYBR Green I as described above. GraphPad Prism (version 7.0) was used to generate dose–response curves by fitting a nonlinear regression curve to the data and then get the best-fit IC_50_ values. The Giemsa-stained thin blood smears of *B. duncani* after garlic oil, black pepper oil, DADS, BCP, atovaquone, and azithromycin exposure at 2× IC_50_ concentration for morphology observation using BZ-X710 All-in-One fluorescence microscope (KEYENCE, Inc., Itasca, IL, USA).

### 4.4. Subculture Studies to Assess Viability of B. Duncani Treated with Garlic Oil, DADS, Atovaquone, Azithromycin, and Their Combinations 

*B. duncani* was treated with garlic oil, DADS, atovaquone, azithromycin, and their combinations at various concentrations in 96-well plate culture system as described above. For garlic oil and DADS, the treated concentrations used were 1×, 1.25×, 1.50×, and 1.75× IC_50_ value; For atovaquone and azithromycin, the treated concentrations used were 1×, 2×, 4×, 8× IC_50_ value. After 72 h treatment, treated RBCs were washed three times in HaRGM and then inoculated in fresh hamster RBCs at the ratio of 1:5. Subcultures were cultured in 96-well plate containing 200 μL of infected hamster RBCs at 2% parasitemia and 2.5% hematocrit each well at 37 °C in a chamber with an atmosphere of 5% CO_2_ with 95% humidity. Subsequently, 1 μL of RBCs from each well was taken and stored at −80 ℃ every one or two days until erythrocytes or controlled uninfected erythrocytes began to lyse. The growth of the subculture was examined by SYBR Green I stain, and the fluorescence units of day 0 were normalized as 0.

## Figures and Tables

**Table 1:** The top 10 active essential oils that showed more than 50% inhibitory effect at 0.001% (*v*/*v*) on *Babesia duncani* growth after 72 h exposure.

Essential Oils and Control Drugs	Plants	Inhibition (%)
Garlic	*Allium sativum*	87
Black pepper	*Piper nigrum*	64
Tarragon	*Artemisia dracunculus*	57
Palo santo	*Bursera graveolens*	56
Coconut oil	*Cocos nucifera*	55
Pine oil	*Pinus sylvestris* L.	53
Meditation	Synergy blend of lavender, cedarwood atlas, tangerine, bergamot, palo santo, patchouli, vetiver, lemon, clove bud, ylang ylang, lime, Peru balsam, cedarwood Virginia, cistus, and chamomile	53
Cajeput	*Melaleuca cajuputi*	53
Moringa oil	*Moringa oleifera*	52
Stress relief	Synergy blend of essential oils of bergamot, patchouli, sweet orange, ylang ylang, pink grapefruit, and gurjum	51
Atovaquone		100
Azithromycin		88

## Supplementary Materials

The following are available online at https://www.mdpi.com/2076-0817/9/6/466/s1, Table S1: Evaluation of a panel of 97 essential oils at a concentration of 0.001% (*v*/*v*) for inhibitory activity against *B. duncani*.
