# Supplementary material for: Identification of Essential Oils Including Garlic Oil and Black Pepper Oil with High Activity against Babesia duncani
Source: Pathogens. 2020 Jun 12;9(6):466. doi: 10.3390/pathogens9060466 (PMC7350376; doi:10.3390/pathogens9060466)
Supplement: Supplementary file 1 [file pathogens-09-00466-s001.pdf]

Table S1. Evaluation of a panel of 97 essential oils at concentration of 0.001% (v/v) for inhibitory activity against *B. duncani*.

No.	EOs (0.001%)	Growth (%)
1	Garlic	13
2	Black pepper	36
3	Tarragon	43
4	Palo santo	44
5	Coconut oil	45
6	Pine oil	47
7	Meditation	47
8	Cajeput	47
9	Moringa oil	48
10	Stress relief	49
11	Ginger	51
12	Rosemary	52
13	Mandarin	52
14	Pink grapefruit	52
15	Alive	53
16	Spruce	53
17	Geranium bourbon	53
18	Neroli	53
19	Sandalwood oil	54
20	Bandit	54
21	Perilla essential oil	55
22	Ravensara	56
23	Cypress	57
24	Vanilla	57
25	Yarrow	58
26	Myrtle	59
27	Bergamot	59
28	Breathe	60
29	Tangerine	61
30	Basil	62
31	Agarwood oil	62
32	Allspice	63
33	Lavender	63
34	Helichrysum oil	64
35	Turmeric	65
36	Nutmeg	65
37	Lemongrass	67

38	Ylang ylang	67
39	Pennyroyal oil	69
40	Anise star	69
41	Oregano	69
42	Hydacheim	69
43	Deep forest	70
44	Eucalyptus	70
45	Davana oil	71
46	Clove bud 2	72
47	Copaiba	73
48	Frankincense	74
49	Lemon	75
50	Dillweed	75
51	Caraway	76
52	Balsam fir	77
53	Cedarwood atlas	78
54	Orange (Sweet)	78
55	Chamomile (German)	78
56	Lime	79
57	Rosewood oil	80
58	Peppermint	81
59	Hyssop	82
60	Coriander oil	82
61	Fir needle	83
62	Star anise oil	83
63	Amyris	85
64	Cumin	85
65	Bay oil	85
66	Citrus blast	87
67	Elemi	88
68	Sage oil	88
69	Vetiver	88
70	Patchouli (Dark)	90
71	Deep muscle	90
72	Juniper Berry	90
73	Fennel sweet	92
74	Citronella	93
75	Lemongrass	94
76	Palmarosa	95
77	Myrrh	96
78	Birch	98
79	Cinnamon bark	98
80	Petitgrain	98

81	Litsea cubeba	99
82	Parsley seed	100
83	Cornmint	101
84	Thyme white	101
85	Wintergreen	104
86	Head ease	106
87	Happy	107
88	Ho wood	109
89	Clary sage	109
90	New beginning	111
91	Tea tree	111
92	Marjoram (Sweet)	113
93	Carrot seed	114
94	Spearmint	114
95	Camphor	124
96	Sleep tight	126
97	Lemon eucalyptus	130
